# Cost-effectiveness of Increasing Access to Contraception during the Zika Virus Outbreak, Puerto Rico, 2016

**DOI:** 10.3201/eid2301.161322

**Published:** 2017-01

**Authors:** Rui Li, Katharine B. Simmons, Jeanne Bertolli, Brenda Rivera-Garcia, Shanna Cox, Lisa Romero, Lisa M. Koonin, Miguel Valencia-Prado, Nabal Bracero, Denise J. Jamieson, Wanda Barfield, Cynthia A. Moore, Cara T. Mai, Lauren C. Korhonen, Meghan T. Frey, Janice Perez-Padilla, Ricardo Torres-Muñoz, Scott D. Grosse

**Affiliations:** Centers for Disease Control and Prevention, Atlanta, Georgia, USA (R. Li, K.B. Simmons, J. Bertolli, S. Cox, L. Romero, L.M. Koonin, D.J. Jamieson, W. Barfield, C.A. Moore, C.T. Mai, L.C. Korhonen, M.T. Frey, J. Perez-Padilla, S.D. Grosse);; Puerto Rico Department of Health, San Juan, Puerto Rico (B. Rivera-Garcia, M. Valenica-Prado, R. Torres-Muñoz);; University of Puerto Rico and Puerto Rico Section of the American College of Obstetricians and Gynecologists, San Juan (N. Bracero)

**Keywords:** contraception, unintended pregnancy, cost-effectiveness, cost-saving, Zika virus, outbreak, microcephaly, viruses, Puerto Rico, vector-borne infections

## Abstract

We modeled the potential cost-effectiveness of increasing access to contraception in Puerto Rico during a Zika virus outbreak. The intervention is projected to cost an additional $33.5 million in family planning services and is likely to be cost-saving for the healthcare system overall. It could reduce Zika virus–related costs by $65.2 million ($2.8 million from less Zika virus testing and monitoring and $62.3 million from avoided costs of Zika virus–associated microcephaly [ZAM]). The estimates are influenced by the contraception methods used, the frequency of ZAM, and the lifetime incremental cost of ZAM. Accounting for unwanted pregnancies that are prevented, irrespective of Zika virus infection, an additional $40.4 million in medical costs would be avoided through the intervention. Increasing contraceptive access for women who want to delay or avoid pregnancy in Puerto Rico during a Zika virus outbreak can substantially reduce the number of cases of ZAM and healthcare costs.

Zika virus infection during pregnancy can cause microcephaly with severe brain damage in the fetus (referred to here as Zika virus–associated microcephaly [ZAM]) and is linked to pregnancy loss and to problems in infants, including eye defects, hearing loss, and impaired growth (*1*). Zika virus is a flavivirus transmitted primarily by infected *Aedes* species mosquitos (*2*). Zika virus can also be sexually transmitted (*3*). Puerto Rico has the largest number of Zika virus disease cases in the United States and its territories (*4*) and, based on extrapolations from the experiences of other countries with Zika virus outbreaks, will probably experience large numbers of Zika virus–exposed pregnancies (*5*).

A primary strategy to reduce Zika virus–associated adverse pregnancy outcomes is to assist women who want to delay or avoid pregnancy. An estimated 65% of pregnancies in Puerto Rico are unintended (unwanted or mistimed), compared with 45% in the continental United States (*2*,*6*). Women in Puerto Rico face multiple barriers to contraceptive use, including high out-of-pocket costs, a shortage of contraceptive supplies, lack of education about options, and a limited number of family planning delivery sites (*2*).

In response to the Zika virus outbreak, the Centers for Disease Control and Prevention and other federal and local partners are seeking to improve access to contraception for women in Puerto Rico who desire it but encounter barriers to accessing the full range of contraception methods, including long-acting reversible contraceptives (LARCs). The objective of this analysis was to estimate the potential cost-effectiveness of increasing access to contraception in Puerto Rico during the 2016 Zika virus outbreak.

## Methods

We constructed a decision tree cost-effectiveness model for a target population of 163,000 women who at the time of the intervention are sexually active with a male partner, fertile, not desiring pregnancy within the next 12 months, and not using permanent contraception methods (e.g., tubal ligation and vasectomy) (Technical Appendix Table and Figure 1). In the no intervention scenario, no changes in contraceptive use distributions from the status quo are expected to occur. In the intervention scenario, women in Puerto Rico are assumed to have same-day access to contraception methods, including LARC, with no out-of-pocket costs. In addition, healthcare providers would be trained to provide client-centered contraceptive counseling and outreach so that women have the information they need to make an informed choice on the contraception method that is best for them. The model specifies contraceptive method use distribution, unintended pregnancy events, and the frequency of ZAM (Technical Appendix Figure 1).

We assumed an intervention in place throughout a year-long Zika virus outbreak in Puerto Rico. We evaluated the costs and outcomes of increased access to contraception compared with no intervention (i.e., status quo). Output measures included numbers of ZAM cases prevented, including stillbirths, elective terminations, and live-born infants, and healthy life years (HLY) gained. Economic benefits of the intervention included avoided costs from ZAM cases prevented and costs avoided for monitoring for Zika virus–exposed pregnancies and infants born from Zika virus–infected mothers. In addition, the avoided cost of prenatal, delivery, postpartum, and neonatal care associated with avoided unwanted pregnancies was considered an economic benefit. In cost-effectiveness analyses, if total avoided cost exceeds the cost of an intervention that improves health, the intervention is considered cost-saving. For scenarios with positive net costs, we reported the incremental cost-effectiveness ratio (ICER), which is the net cost per HLY gained in comparison to the status quo.

Independent of Zika virus–exposed pregnancies and ZAM, unintended pregnancy is associated with adverse maternal and child health outcomes. Because roughly 60% of unintended pregnancies are classified as mistimed, which might result in a delayed rather than avoided pregnancy, with the same costs occurring later (*7*), we only estimated avoided medical costs from prevention of the 40% of unintended pregnancies presumed to be not desired at a later time irrespective of Zika virus infection. However, we included all ZAM cases prevented during the intervention period.

### Contraception Use with and without the Intervention

We estimated the inputs for the decision-tree model and their sources (Table 1). In the no intervention scenario, we took the distribution of women in the target population by use of different types of reversible contraceptives (or no use) from a 2002 survey administered in Puerto Rico and adjusted it to reflect the 36% decrease in fertility rates in Puerto Rico during 2002–2015 (*8*,*23*,*24*).

**Table 1:** Estimates of input parameters used in a model to assess cost-effectiveness of increasing access to contraception to women during the Zika virus outbreak, Puerto Rico, 2016*

Parameter	Value in main scenario (range)	Distribution	Source
Epidemio parameters			
Target population size†	163,000		
Contraceptive use distribution at baseline‡			(*8*)
No method	9.35%		
Less-effective methods	44.77%		
Moderately effective methods	33.93%		
Dual-method use with condoms	9.36%		
Highly effective methods	2.60%		
% Women receiving contraceptive services with intervention			NSFG 2011–2013, expert opinion
No contraception users§	50% (30%–70%)	Uniform	
Less-effective method users§	60% (30%–80%)	Uniform	
Moderately effective method users§	100%		
% Women switching to a more effective method after receiving contraception counseling	50% (10%–80%)	Uniform	In-house expert opinion
Among women switching to a more effective method, % choosing highly effective methods¶	50% (33%–67%)	Uniform	(*9*), authors’ assumptions
Contraceptive failure rate over 1 year			(*10*)
No method	85% (68%–100%)	Triangular	
Less-effective methods	22% (18%–27%)	Triangular	
Moderately effective methods	9% (7%–11%)	Triangular	
Dual-method use with condoms	1.2% (1.0%–1.4%)	Triangular	Derived from NSFG 2011–2013; K. Pazol, pers. comm., ONDIEH, CDC, 2016
Highly effective methods	0.5% (0.4%–0.6%)	Triangular	
Distribution of outcomes of unintended pregnancies			
Induced abortion	28%		Calculated based on (*11*)
Spontaneous abortion/fetal death	14%		(*12*)
Live birth	58%		Calculated based on (*11*) and (*12*)
Prevalence of Zika virus infection	25% (10%–70%)	Uniform	(*12*)
Prevalence of ZAM among mid-trimester pregnancies#	58/10,000 (32/10,000–86/10,000)	Uniform	(*5*)
Stillbirth rate of fetus with microcephaly	7% (5.4%–8.4%)	Triangular	(*13*)
Termination rate of fetus with ZAM**	28% (20%–50%)	Uniform	(*14*)
HLY lost because of 1 case of ZAM	30		(*15*)
Cost parameters, in 2015 US dollars			
Intervention: training physicians, outreach, and administrative cost††	$39 ($31–$47)	Triangular	Budget from a pilot program to increase contraception access in Puerto Rico during 2016 Zika outbreak
Contraceptive counseling	$10 ($5–$25)	Uniform	
Contraceptive methods and related services††,‡‡			(*16*)
Highly effective contraceptive methods§§	$666 ($533–$799)	Triangular	
Moderately effective contraceptive methods¶¶	$417 ($334–$501)	Triangular	
Dual-method use##	$452 ($362,$543)	Triangular	
Less-effective contraceptive methods***	$35 ($28–$42)	Triangular	
LARC insertion†††	$165 ($132–$198)	Triangular	(*16*)
LARC removal	$109 ($87–$131)	Triangular	(*16*)
Provider office visit (for moderately effective method users)	$43 ($34–$51)	Triangular	(*16*)
Provider office visit (for highly effective method users)	$104 ($83–$125)	Triangular	(*16*)
Prenatal, delivery and postpartum care for mother and neonatal care for infant (not Zika virus–related)††,‡‡‡	$22,067 ($17,652–$26,479)	Triangular	Weighted average of vaginal and C-section from (*17*)
Prenatal care	$3,506		
Delivery and postpartum care	$10,960		
Neonatal care	$7,599		
Induced abortion††	$1,100 ($880–$1,320)	Triangular	Derived from 2014 MarketScan Commercial Claims database
Spontaneous abortion††	$1,100 ($880–$1,320)	Triangular	Assumed same as for induced abortion
Mid-trimester pregnancy termination††	$2,725 ($2,180–$3,269)	Triangular	(*18*)
Stillbirth††	$5,007 ($4,006–$6,009)	Triangular	(*15*)
Zika virus–associated cost			
Cost of Zika-associated testing and monitoring of women during pregnancy††,§§§	$439 ($351–$527)	Triangular	Derived from 2014 MarketScan commercial claims database
Cost of Zika-associated testing among live-born infants with Zika-infected mothers††,¶¶¶	$211 ($169–$253)	Triangular	Derived from MarketScan commercial claims database, 2009–2014
Cost of testing for fetus with ZAM††,###	$330 ($264–$396)	Triangular	Derived from MarketScan commercial claims database, 2009–2014
Cost of stillbirth with ZAM††,****	$5,776 ($4,621–$6,931)	Triangular	(*15*)
Cost of termination of fetus with ZAM††,††††	$5,027 ($4,021–$6,032)	Triangular	(*17**,**18*)
Cost of live-born infant with ZAM††,‡‡‡‡	$22,715 ($18,172–$27,258)	Triangular	(*17*)
Lifetime direct cost of live-born infants with ZAM§§§§	$3,788,843 ($2,243,124–$5,545,652)	Triangular	Derived in part from MarketScan commercial claims database, 2009–2014

*BRFSS, Behavior Risk Surveillance System; CDC, Centers for Disease Control and Prevention; HLY; healthy life years; IUD, intrauterine device; LARC, long-acting reversible contraceptive; NSFG, National Survey of Family Growth; ONDIEH, Office of Noncommunicable Diseases, Injury and Environmental Health; ZAM, Zika virus–associated microcephaly. †Authors’ calculation based on 2015 Puerto Rico total population size, 2015 birth rate, and adjusted contraception usage in 2002 BRFSS in Puerto Rico (online Technical Appendix Table, http://wwwnc.cdc.gov/EID/article/23/1/16-1322-Techapp1.pdf). ‡Less-effective methods include condoms, spermicides, fertility awareness methods, withdrawal, sponge, and diaphragm. Moderately effective methods include oral contraceptive pills, patches, vaginal rings, and injectable contraceptives. Highly effective method includes IUDs and subdermal implants. Dual use refers to the combination of moderately effective method use and male condoms. The contraception use distribution at baseline (without intervention) is based on adjusting the distribution reported in the 2002 BRFSS in Puerto Rico excluding women using permanent contraception by assuming 5 percentage points fewer women using no contraception than in 2002, which is based on a 36% reduction of birth rate among women 15–44 years of age in Puerto Rico from 2002 to 2015 (National Vital Statistics Report for 2002 and 2015 unpublished birth data from Puerto Rico), and the reported reasons for US teen pregnancy reduction (http://www.cdc.gov/nchhstp/newsroom/docs/factsheets/yrbs-fact-sheet-final-508.pdf); the decline in teen pregnancies was the fastest of any age group in Puerto Rico during 2010–2014, as reported in National Vital Statistics Reports. Data from Title X clinics in Puerto Rico also show that the percentage of women of reproductive age served in Title X clinics increased from 2.2% in 2006 to 10% in 2015. We also assume that new contraception users have the same contraception method distribution as contraception users as reported in the 2002 BRFSS survey (*8*). We assume 22% dual use among moderately effective method users at baseline (based on NSFG 2011–2013 data and unpublished analyses supplied by Karen Pazol, ONDIEH, CDC; we accounted for the effectiveness of dual-method use in preventing pregnancy but not for preventing sexual transmission of Zika virus). We calculated method-specific annual pregnancy rates by multiplying the failure rates of contraception methods under typical use by a calculated correction factor of 0.88 to adjust for the model over-predicting the number of unintended births in 2015 using the typical failure rates only. Multiplying method-specific contraceptive failure rates by numbers of women in each method category typically results in more predicted pregnancies and births than are actually observed, in part because of heterogeneity in sexual behaviors (*19*). §Assuming 50% of no contraception users, 60% of less-effective contraception users, and 100% of moderately effective contraception users will visit a healthcare provider for contraception services under intervention. Based on the NSFG 2011–2013, among women of reproductive age who did not intend to become pregnant and not using permanent contraceptive methods, 21% of no contraception users, 33% less-effective method users, 97% moderately effective method users had at least 1 visit for contraception services in the last 12 months (personal communication, Karen Pazol, ONDIEH, CDC, 2016). Contraceptive services include receiving a birth control method or a prescription, receiving a checkup for birth control, receiving counseling about birth control, receiving a sterilizing operation, receiving counseling about a sterilizing operation, receiving emergency contraception, or receiving counseling about emergency contraception. We assume the intervention will increase the percentage of women visiting their provider for contraception counseling. ¶In the Contraceptive CHOICE project (*9*), 67% of participants who wished to avoid pregnancy chose to use highly effective methods, and 33% chose to use moderately effective methods. For the main scenario, we applied those estimates to 40% of the target population, assuming that 40% of unintended pregnancies are unwanted, and assumed that 20% of the remaining 60% of switchers would choose highly effective methods. #Ellington et al. (*5*) estimated 180 cases (interquartile range 100–270 cases) of congenital microcephaly associated with Zika virus in Puerto Rico among an estimated 31,272 births (unpublished birth data, Puerto Rico Department of Health, 2015). On the basis of those estimates, we estimated the prevalence of congenital microcephaly: 58/10,000 births (interquartile range 32/10,000–86/10,000). **The pregnancy loss rate in the US Zika Pregnancy Registry as of July 21, 2016, was 35% (*14*). The termination rate is calculated as the overall pregnancy loss rate minus the assumed stillbirth rate. ††A range of the main value ±20% was used to create upper and lower bounds used in sensitivity analyses. ‡‡Includes cost for devices and costs for 1 year of injections, pills, patches, rings, condoms, and the cost for related services in the first year. §§Weighted average cost for hormonal IUD (50%, $659), copper IUD (25%, $598), and implant (25%, $659), based on Contraception CHOICE study (*9*) and commonly used devices in Puerto Rico. ¶¶Weighted average cost for generic contraceptive pill (78%, $370/y), injectable (14%, $240/y plus $130 for consultation for the year), and ring or patch (8%, $964/y), with the distribution of moderately effective methods based on NSFG, 2011–2013. ##Weighted average cost of moderately effective method plus cost of less-effective method. ***Male condom. †††Including the cost for checking the placement of IUD in the first year of insertion. ‡‡‡Weighted average for vaginal and C-section delivery, including prenatal care, delivery, postpartum, and neonatal cost at delivery and in the first 3 months. §§§Assumes additional costs related to repeated Zika virus testing by IgM (1–2 tests) for all pregnant women, 4 extra detailed ultrasound examinations, and 25% of women getting amniocentesis during all Zika-positive pregnancies (25% infection rate at baseline, range 10%–70%) (*5*), based on Oduyebo et al. (*20*). ¶¶¶Assumes 2 Zika virus tests (IgM and PCR) for serum and placenta, cranial ultrasound, and eye examination for all infants born to Zika virus–positive mothers based on Fleming-Dutra et al. (*21*). ###Three PCR tests for Zika virus using placenta, cord, and brain tissues of the fetus based on Martines et al. (*22*). ****Assuming all prenatal care cost, including extra cost of Zika virus–associated testing and monitoring during pregnancy and extra cost for Zika virus–associated testing for fetus. ††††Assuming half of the prenatal care cost, including extra cost of Zika virus–associated testing and monitoring during pregnancy and extra cost for Zika virus–associated testing for fetus. ‡‡‡‡Cost of prenatal care and delivery and extra cost of Zika virus–associated testing and monitoring during pregnancy and testing of infants for Zika virus. §§§§Present value of cumulative medical and supportive care cost for infant with ZAM, discounted at 3% annually and taking into account mortality. Expenditures by employer-sponsored health plans for privately insured children with combined diagnoses of microcephaly and congenital cytomegalovirus enrolled during the first 4 years of life were used to project medical costs for cases of ZAM.

For the main intervention scenario, we assumed that 50% of no contraception users, 60% of less-effective contraceptive method users, and 100% of moderately effective contraceptive method users would visit a healthcare provider during the intervention period and be counseled about contraception use (Table 1). The first 2 percentages are roughly twice the percentages of women reported in the 2011–2013 US National Survey on Family Growth to have received contraceptive services (contraception or counseling) within the past year because we assumed that, during the Zika virus outbreak, more women and providers would discuss contraception; virtually all moderately effective method users were assumed to see providers to obtain contraceptive prescriptions.

For the main scenario, we also assumed, optimistically, that 50% of women in the target population who receive contraceptive services during the Zika virus outbreak would be willing to change to a more effective contraceptive method, evenly divided between moderately effective and highly effective methods. We applied data from the Contraceptive CHOICE Project (67% of participants used LARC and 33% used moderately effective methods) (*9*) to the 40% of women assumed to not want to be pregnant; we assumed 20% of other women not intending pregnancy would use LARC. We further assumed that 30% of moderately effective contraception users would also choose to use condoms (dual-method use) under the intervention, based on a study reporting dual-method use among persons at risk for HIV (*25*).

### Epidemiologic Model Input Parameters

We calculated method-specific annual pregnancy rates by applying failure rates of contraception methods under typical use (*10*), in combination with information on estimated numbers of unintended pregnancies, to adjust for other factors influencing pregnancy risk (*19*). We estimated the proportion of fetal losses among unintended pregnancies from data for the Caribbean region, including Puerto Rico (*12*), and calculated the proportion of induced abortion among unintended pregnancies from a survey conducted in Puerto Rico in 2001 (the latest year for which data were available) (*11*). We assumed that the distribution of fetal loss and induced abortions in unintended pregnancies unaffected by ZAM would not be altered by the Zika virus outbreak or the intervention.

For adverse pregnancy and birth outcomes associated with Zika virus, we only considered ZAM and associated brain anomalies, including live births, stillbirths, and terminations attributable to prenatal diagnosis. Although Zika virus can cause brain lesions and dysfunction in fetuses and newborns who do not have microcephaly (*26*), we lacked the data to model their prevalence and cost. In the main analysis, we assumed 58 cases of ZAM per 10,000 live births (range 32–86/10,000) based on a modeling study that considered data from other mosquitoborne illnesses in Puerto Rico and Zika virus outbreaks in other locations (*5*). We assumed a pregnancy loss rate of 35% among Zika virus–exposed fetuses with diagnosed birth defects based on cases in the US Zika Pregnancy Registry as of July 21, 2016 (*14*).

A summary measure of population health impact is healthy life expectancy at birth. We projected gains in HLY by multiplying total cases of ZAM prevented by 30.0, which is the average number of quality-adjusted life-years at birth in the United States for an infant without severe microcephaly (*15*) and the estimated loss in disability-adjusted life years from microcephaly (*27*). We multiplied 30.0 by the sum of live births and fetal losses associated with ZAM to calculate gains in HLY. We included fetal losses in the HLY calculations because in the absence of ZAM those pregnancies would have resulted in live births, with the same healthy life expectancy as other children (*15*).

### Cost Parameters

We conducted the analysis from a healthcare system perspective that includes direct medically related costs regardless of payer. We used payments from private insurance because payments from Medicaid might underestimate the cost of healthcare (*28*). Intervention costs included program costs of training providers, patient educational materials, outreach/media campaigns on the availability of contraceptives services, and program coordination and the incremental costs of family planning services. The latter comprised the costs of contraception methods and related office visits and services (e.g., insertion and removal of LARC for new method users resulting from the intervention and the cost of more intensive counseling for all women receiving contraceptive services during the intervention). We took the 1-year costs for contraception methods from the literature (*16*,*29*) and based the other program costs on the estimated costs for a pilot program planned to increase access to contraception in Puerto Rico as part of the current Zika virus outbreak response (*30*). We did not apply a discount rate to intervention costs because of the time horizon of 12 months.

Zika virus–related costs prevented by this intervention were in 2 parts: 1) costs for Zika virus testing and monitoring for Zika virus–exposed pregnancies and infants, and 2) costs of ZAM cases (Table 1). The cost estimates for testing and monitoring presumed 100% adherence by clinicians and patients to recommendations (*20*–*22*).

The lifetime cost per live-born infant with ZAM includes direct medical and nonmedical costs. ZAM is among the most severe types of microcephaly and is associated with loss of brain tissue volume, increased fluid spaces, and intracranial calcifications. All 3 cases of live-born infants with ZAM in French Polynesia demonstrated severe neurologic outcomes with delayed cognitive development (*26*). On the basis of expert opinion, infants with ZAM who survive the neonatal period would be expected to have neurologic dysfunction consistent with severe cerebral palsy within 1–2 years of birth.

As a proxy for the medical cost of ZAM, we used the estimated cost of treating infants with microcephaly associated with a diagnosis of symptomatic congenital cytomegalovirus (CMV). We used the MarketScan Commercial Database (Truven Health Analytics) with a sample of ≈100 million US residents covered by employer-sponsored insurance at any time during 2009–2014. We used average costs for 4 newborn infants with diagnoses of microcephaly and CMV who survived and were enrolled in a health plan for >3 years. For the direct nonmedical cost of ZAM, we used the estimated cost for supportive care for children with severe congenital brain injury, both paid care and unpaid care. The total lifetime cost for surviving infants with ZAM was estimated at $3.8 million per infant, taking into account infant and child mortality and discounting of costs in future years at a 3% rate per year; the sum of undiscounted costs for children who survive to adulthood might reach $10 million.

We determined the estimated non–Zika virus–related medical costs associated with women’s prenatal care, labor and delivery, and postpartum care for pregnancies ending in live birth and neonatal care from a study of US commercial health plan expenditures (*17*). Estimates for costs associated with pregnancies ending in induced abortion were based on our analyses of commercial claims data (Table 1).

### Sensitivity Analyses

Because many parameters used in the model are uncertain, we conducted sensitivity analyses on selected parameters, including different scenarios for the baseline and postintervention contraception use distributions in Puerto Rico. We tested alternate baseline contraception use distributions in Puerto Rico for women at risk for unintended pregnancy by using the actual distribution of method use reported in 2002 (*8*) and among women attending Title X clinics in Puerto Rico in 2014 (*31*). For the postintervention contraception use distribution, we tested scenarios assuming different proportions of women receiving contraceptive services from a healthcare provider, different levels of willingness to switch to a more effective method, and different shares of moderately effective and highly effective methods among switchers. Other parameters evaluated during sensitivity analysis included the incidence of ZAM during the Zika virus outbreak in Puerto Rico, percentage of pregnancies with ZAM terminated, the cost of caring for a live-born infant with microcephaly, and the cost of the intervention.

We conducted sensitivity analyses in which we altered selected assumptions. In one, we annualized the cost of LARC devices considering the expected duration of method use. In another, we adjusted observed data on US healthcare and supportive care costs to the generally lower levels of prices in Puerto Rico market by applying conversion factors of ratios of healthcare spending per capita and wages of nurse assistants between the United States and Puerto Rico (*32*,*33*). We also conducted a probabilistic sensitivity analysis by using Monte Carlo simulation (10,000 draws) that assumed different distributions for all the parameters used in the model (Table 1). All analyses were conducted using TreeAge Pro 2016 software (TreeAge Software, Williamstown, MA, USA) and Excel 2013 (Microsoft, Redmond, WA, USA). All costs were adjusted to 2014 US dollars by using the health component of the Personal Consumption Expenditures price index (*34*).

## Results

In the main scenario, we predict the intervention would prevent 25 cases of ZAM among unintended pregnancies avoided, of which 16 would have resulted in live births (Table 2). The incremental intervention cost of US $33.5 million (i.e., $206 per member of target population) relative to no intervention (status quo) is more than offset by $65.2 million in avoided Zika virus–associated costs, $2.8 million from extra testing and monitoring for pregnant women and infants for Zika virus–exposed pregnancies avoided, and $62.3 million from ZAM cases prevented. The net savings from Zika virus–associated costs alone is $31.7 million.

**Table 2:** Zika virus–associated microcephaly cases and costs, as well as additional costs associated with unwanted pregnancies, with and without intervention to increase access to contraception to women during the Zika virus outbreak, Puerto Rico, 2016, in main scenario*†‡

Parameter	Without intervention	With intervention	Difference
Prevention of ZAM and Zika virus–associated cost			
Total no. ZAM cases	99	74	−25
No. pregnancy terminations	28	21	−7
No. stillbirths	7	5	−2
No. live births	64	48	−16
Cost of family planning services (under intervention also includes program cost)	$38,269,679	$71,738,133	$33,468,454
Total Zika virus–associated cost	$256,578,162	$191,422,342	–$65,155,820
Costs of extra testing and monitoring for Zika virus during pregnancy and for infants exposed in utero during Zika virus outbreak§	$11,125,061	$8,303,158	–$2,821,903
Direct costs of ZAM¶	$245,453,101	$183,119,184	–$62,333,917
Pregnancy terminations	$139,343	$103,956	–$35,387
Stillbirths	$40,025	$29,861	–$10,165
Live births	$245,273,733	$182,985,368	–$62,288,366
Cost savings from Zika virus–associated cost avoided only#			–$31,687,366
Prevention of unwanted pregnancies			
No. of unwanted pregnancies**	11,995	8,949	−3,046
No. induced abortions	3,385	2,525	−860
No. spontaneous abortions and fetal deaths	1,679	1,253	−426
No. unwanted live births	6,856	5,117	−1,739
Medical cost for unwanted pregnancy	$159,074,573	$118,722,504	–$40,352,069
Net cost savings from avoiding both Zika virus–associated cost and unwanted pregnancy cost††			–$72,039,435

*ZAM, Zika virus–associated microcephaly. †The numbers in the columns and rows might not exactly match because of rounding. ‡Target population size: 163,000 women who do not intend to become pregnant during Zika virus outbreak. Women of reproductive age in Puerto Rico who are sexually active with a male partner, fertile, not desiring pregnancy, and not using permanent contraception methods (e.g., tubal ligation and vasectomy). §Only including cost of testing for Zika virus and monitoring for exposed infants without ZAM; testing costs for infants with ZAM are included in the direct costs of ZAM. ¶From healthcare system perspective, includes direct medical and medical-related costs, including supportive care for persons with ZAM, even if the cost might not be paid by healthcare payers or delivered by healthcare providers. #Total Zika virus–associated cost avoided (absolute value) minus the additional cost of family planning service under intervention compared with no intervention. **Unwanted pregnancies which are not desired in the future (assuming 60% of unintended pregnancies are mistimed), irrespective of Zika virus infection ††Absolute value of net medical cost for unwanted pregnancy plus absolute value of net cost savings from Zika virus–associated costs avoided.

The number of ZAM cases prevented and Zika virus–associated costs avoided are sensitive to the proportion of women receiving contraceptive services and the proportion of those women willing to switch to a more effective contraception method during the Zika virus outbreak (Figure; Table 3). If the proportions of women receiving contraception services are assumed to be the same as estimated for the continental United States in the National Survey of Family Growth for 2011–2013 (i.e., 21% among no contraception users, 33% among less-effective method users, and 97% among all moderately effective method users), 16 cases of ZAM are prevented, and the net savings is $15.4 million (Table 3). If 10% of women receiving contraceptive services switch to a more effective method, 6 cases of ZAM are prevented, and net saving is $2.8 million. If the intervention only shifts users of moderately effective methods to a highly effective method (no change in non-use or use of less-effective methods), 7 ZAM cases are prevented, with an ICER of $24,608/HLY gained. Increasing the proportion of dual-method users increases the number of cases of ZAM prevented and net savings attributable to higher contraception effectiveness. The results are also sensitive to the prevalence of ZAM among mid-trimester pregnancies, the percentage of ZAM cases resulting in live-born infants, lifetime cost per live-born infant with ZAM, and the intervention cost. If we adjust US cost estimates for lower prices in Puerto Rico while keeping intervention costs at US prices, net savings are $1.7 million. In all but 1 of the scenarios tested, the intervention is cost-saving.

**Figure:**
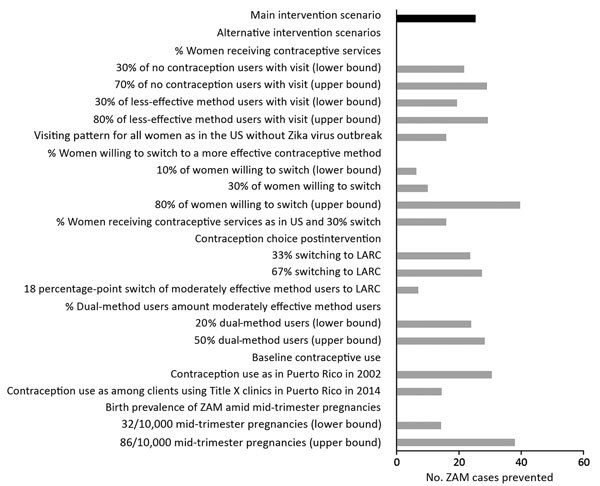
Sensitivity analysis indicating the effect of changes of assumptions on the number of ZAM cases prevented in a proposed intervention to increase access to contraception to women during the Zika virus outbreak, Puerto Rico, 2016. LARC, long-acting reversible contraceptive; ZAM, Zika virus–associated microcephaly.

**Table 3:** Sensitivity analyses indicating the number of ZAM cases prevented and Zika virus–associated costs avoided in proposed intervention to increase access to contraception to women during Zika virus outbreak, Puerto Rico, 2016*

Parameter	No. ZAM cases prevented	Incremental intervention cost, millions	Zika virus–associated cost avoided, millions	Total incremental cost,† millions	Cost per HLY gained	Additional cost avoided from UP, millions
Main scenario	25	$33.5	$65.2	–$31.7	CS	$40.4
% Women receiving contraceptive services from healthcare provider; main scenario, 50% of no method users, 60% of less-effective method users, and 100% of moderately effective method users						
30% of no method users‡	22	$32.4	$55.8	–$23.5	CS	$34.6
70% of no method users	29	$34.6	$74.5	–$39.9	CS	$46.1
30% of less-effective method users	19	$26.0	$50.0	–$24.0	CS	$31.0
80% of less-effective method users	29	$38.5	$75.2	–$36.8	CS	$46.6
% Women receiving contraceptive services as in NSFG 2011–2013§	16	$25.2	$40.6	–$15.4	CS	$25.1
% Women willing to change to more effective method;¶ main scenario value: 50%						
10%	6	$13.0	$15.8	–$2.8	CS	$9.7
30%	16	$23.2	$40.5	–$17.3	CS	$25.0
80%	39	$48.8	$102.2	–$53.3	CS	$63.3
% Women receiving contraceptive services from healthcare provider as in NSFG 2011–2013 with 30% of them willing to change to a new method	10	$18.2	$25.7	–$7.6	CS	$15.9
Use of highly effective methods among switchers; main value 50%						
67%	27	$38.4	$69.9	–$31.5	CS	$43.3
33%	23	$28.5	$60.4	–$31.8	CS	$37.4
Contraception switching pattern reported in Colorado Family Planning Initiative#	7	$21.8	$17.0	$4.8	$24,608	$10.5
Dual-method use; 30% of moderately effective method users in main scenario						
20% of moderately effective users	24	33.1	61.3	–$28.2	CS	$38.0
50% of moderately effective users	28	34.1	−72.9	–$38.7	CS	$45.1
Contraception use distribution at baseline						
As reported in 2002 BRFSS survey**	30	33.6	−78.4	–$44.8	CS	$48.6
As in Title X clinics in 2014††	14	$30.1	$36.7	–$6.6	CS	$22.7
Rate of ZAM among all live-born infants; main scenario value 58/10,000						
32/10,000	14	$33.5	$37.5	–$4.0	CS	$40.4
86/10,000	38	$33.5	$96.3	–$62.8	CS	$40.3
Lifetime costs for microcephaly; main scenario value $3.8 million						
$1.9 million	25	$33.5	$33.5	0	CN‡‡	$40.4
$2.2 million	25	$33.5	$39.5	–$6.1	CS	$40.4
$5.5 million	25	$33.5	$93.5	–$60.0	CS	$40.4
Termination of pregnancy with ZAM						
20%	25	$33.5	$72.8	–$39.3	CS	$40.4
50%	25	$33.5	$44.1	–$10.6	CS	$40.3
Cost of the program other than providing the contraception at no cost to patients; main scenario value $39/person						
$0/person	25	$27.1	$65.2	–$38.0	CS	$40.4
$100/person	25	$43.4	$65.2	–$21.8	CS	$40.4
Annualized LARC device cost	25	$17.5	$65.2	–$47.7	CS	$40.4
Puerto Rico costs§§	25	$30.8	$32.5	–$1.7	CS	$14.4
Discount rate						
0%	25	$33.5	$105.4	–$72.0	CS	$40.4
5%	25	$33.5	$52.9	–$19.4	CS	$40.4

*BRFSS, Behavioral Risk Factor Surveillance System; CN, cost-neutral; CS, cost-saving; HLY, healthy life years; LARC, long-acting reversible contraceptive; NSFG, National Survey of Family Growth; UP, unwanted pregnancy; ZAM, Zika virus–associated microcephaly. †Total incremental cost is the additional cost of contraception minus Zika virus–associated cost avoided. ‡30% of no contraception users, 60% of less-effective contraceptive method users, 100% of moderately effective contraceptive method users seeking contraceptive services from healthcare provider during the Zika virus outbreak. §Based on NSFG 2011–2013, among women of reproductive age who are sexually active, did not intend to become pregnant, and were not using permanent contraceptive methods, 21% of no contraception users, 33% of less-effective contraceptive method users, 97% of moderately effective contraceptive method users, and 94% of dual-method users had at least 1 contraceptive service visit in the last 12 months (in total 50%). ¶Based on Title X Family Planning annual report for 2007–2015 in Colorado, 30% of clients who visited Title X clinics switched to a new method. #Eighteen percentage points of users of moderately effective methods are assumed to switch to highly effective methods, of whom 21% were dual-method users. **Contraception distribution in Puerto Rico in 2002 15.9% no method, 41.6% less-effective methods, 40.2% moderately effective methods, and 2.4% highly effective methods. ††In 2014, in Title X clinics in Puerto Rico, 20% of women at risk for unintended pregnancy used less-effective methods, 77% used moderately effective methods, and 2% used highly effective methods. ‡‡Intervention cost equals to the medical savings from ZAM cases prevented. §§Conversion factor of 0.36 applied to pregnancy and ZAM medical costs based on the ratio of per capita medical expenditure in Puerto Rico and in the United States in 2012 as in Portela et al. 2015 (*32*); conversion factor of 0.72 applied to costs of supportive care for live-born infants with ZAM, based on the ratio of annual salary for assistant nurses in Puerto Rico and in the United States (*33*).

A probabilistic sensitivity analysis scatter graph shows that most of the model simulations result in ICERs in the lower right quadrant with lower costs and better health outcomes (Technical Appendix Figure 2). Specifically, the intervention is cost-saving in 92.11% of the 10,000 iterations, and in 98.10% of the iterations, the intervention has an ICER of <$20,000/HLY gained.

The intervention is also predicted to prevent $40.4 million in medical costs from unwanted pregnancies avoided in the main scenario (Table 2). In many sensitivity analyses, the cost avoided from these unwanted pregnancies prevented alone is greater than the intervention cost. The larger the numbers of no contraception users and less-effective method users receiving contraceptive services and willing to switch to more effective methods, the greater the magnitude of cost savings from unwanted pregnancies avoided (Table 3).

## Discussion

The results of our modeling analysis suggest that increasing access to effective contraception in the context of the 2016 Zika virus outbreak for women in Puerto Rico who do not intend to become pregnant could proportionally reduce the number of unintended pregnancies and cases of ZAM by 25%. The intervention is cost-saving (negative net cost) when considering the benefits from preventing ZAM and avoiding Zika virus–exposed pregnancy costs in the main scenarios and in most of the scenarios we tested. In scenarios in which the intervention is not cost-saving, it is still cost-effective relative to accepted cost-effectiveness thresholds (*35*). The World Health Organization suggests that interventions that cost <3 times the gross domestic product per capita per HLY (equivalent to $150,000 in the United States and $60,000 in Puerto Rico) are cost-effective and those costing less than gross domestic product per capita are highly cost-effective (*36*). When considering additional benefits from preventing unintended pregnancies not desired at a later time, the intervention is cost-saving in all scenarios. Previous studies have shown that expanding access to contraception, especially LARC, is cost-saving (*16*,*37*,*38*). Likewise, our findings suggest that this intervention could be cost-saving or cost-effective within the context of a public health emergency response.

Our study has several limitations. First, we project the effects of a hypothetical intervention in place in Puerto Rico during the 2016 Zika virus outbreak. However, the qualitative results would apply in future outbreaks. Second, the baseline contraception use distribution is based on a 2002 survey; the current distribution in Puerto Rico might be different. Third, uncertainty exists about the effect of the proposed intervention on postintervention contraceptive use distribution; however, the sensitivity analyses indicate that different distributions of LARC types among switchers does not have a substantial influence on the results. Fourth, our study assumes that women have full access to healthcare providers. In areas with limited access to providers, the effectiveness of the intervention might be lower, although Puerto Rico has a similar ratio of physicians to population as the United States as a whole (*39*), and despite a loss of physicians in recent years, Puerto Rico has a network of providers, federally qualified health clinics, and Title X providers in rural and urban areas. Fifth, the distribution of outcomes of unintended pregnancies in Puerto Rico is uncertain. We lack data on miscarriage and induced abortion rates in Puerto Rico and so did not have sufficient data to model uncertainty in these parameters. The rates of stillbirth and pregnancy termination among pregnancies with ZAM in Puerto Rico are also unknown. Our assumed percentage of live births among pregnancies with recognized ZAM (65%) compares with a 38% rate reported in French Polynesia during the 2013 Zika virus outbreak (*11*). Sixth, pregnancy intentions and use of contraception among women in Puerto Rico might differ during the Zika virus outbreak compared to preoutbreak periods. Seventh, our analysis does not consider possibly higher rates of fetal loss and induced abortion among women infected by Zika virus during early pregnancy or brain abnormalities or conditions related to Zika virus not involving microcephaly. Eighth, the assumed Zika virus testing costs assume 100% adherence to recommended testing practices; the actual cost savings taking nonadherence into account would be lower. Ninth, the cost estimates of ZAM cases in live-born infants do not include costs of managing mental health conditions among parents of affected infants. Tenth, using private insurance payments might overstate the healthcare cost of treating ZAM. However, if the cost of ZAM exceeds $1.9 million, the intervention is still cost-saving. Finally, if efforts to prevent transmission of Zika virus in Puerto Rico are effective, the rate of infection in pregnancy and the incidence of ZAM relative to that projected could be reduced.

Despite its limitations, our study has several strengths. First, the study is based on the most current available information. Second, the contraception scenarios are based on real-world programs and have resulted from consultation with subject matter experts. Third, expenditure data from a large sample of US residents with commercial health insurance were used to calculate the potential medical cost of ZAM on the basis of combinations of diagnostic codes for virus-associated microcephaly, although costs might be lower for similar children with public insurance. Finally, sensitivity analyses give consistent results indicating expected net cost savings associated with an intervention that would increase access to contraception in response to the Zika virus outbreak in Puerto Rico.

Zika virus can cause devastating birth defects, and infants born with ZAM and their families will require lifelong support. Avoiding unintended pregnancies is a critical intervention to mitigate the effects of ZAM. Efforts to prevent adverse Zika virus–related pregnancy outcomes in Puerto Rico are especially important because of limited resources (*40*). Our analyses suggest that increasing access to a full range of contraception among women in Puerto Rico who want to delay or avoid becoming pregnant during a Zika virus outbreak would be a cost-saving strategy to reduce the effects of ZAM. The magnitude of cost savings is even greater when considering the avoided cost of unwanted pregnancies prevented.

Technical Appendix Process for deriving the size of the target population, decision tree structure, and probabilistic sensitivity analysis of cost-effectiveness for a proposed intervention to increase access to contraception to women during the Zika virus outbreak, Puerto Rico, 2016.

## References

[1] Rasmussen SA, Jamieson DJ, Honein MA, Petersen LR. Zika virus and birth defects—reviewing the evidence for causality. N Engl J Med. 2016;374:1981–7. 10.1056/NEJMsr1604338 27074377

[2] Tepper NK, Goldberg HI, Bernal MI, Rivera B, Frey MT, Malave C, et al. Estimating contraceptive needs and increasing access to contraception in response to the Zika virus disease outbreak—Puerto Rico, 2016. MMWR Morb Mortal Wkly Rep. 2016;65:311–4. 10.15585/mmwr.mm6512e1 27031817

[3] Oster AM, Russell K, Stryker JE, Friedman A, Kachur RE, Petersen EE, et al. Update: interim guidance for prevention of sexual transmission of Zika virus—United States, 2016. MMWR Morb Mortal Wkly Rep. 2016;65:323–5. 10.15585/mmwr.mm6512e3 27032078

[4] Centers for Disease Control and Prevention. Zika virus disease in the United States, 2015–2016 [cited 2016 May 3]. http://www.cdc.gov/zika/geo/united-states.html

[5] Ellington SR, Devine O, Bertolli J, Martinez Quiñones A, Shapiro-Mendoza CK, Perez-Padilla J, et al. Estimating the number of pregnant women infected with Zika virus and expected infants with microcephaly following the Zika outbreak in Puerto Rico, 2016. JAMA Pediatr. 2016 Aug 19 [Epub ahead of print]. 10.1001/jamapediatrics.2016.2974 27544075

[6] Finer LB, Zolna MR. Declines in unintended pregnancy in the United States, 2008–2011. N Engl J Med. 2016;374:843–52. 10.1056/NEJMsa1506575 26962904 PMC4861155

[7] Trussell J. Overstating the cost savings from contraceptive use. Eur J Contracept Reprod Health Care. 2008;13:219–21. 10.1080/13625180802359263 18821460

[8] Bensyl DM, Iuliano DA, Carter M, Santelli J, Gilbert BC. Contraceptive use—United States and territories, Behavioral Risk Factor Surveillance System, 2002. MMWR Surveill Summ. 2005;54:1–72. 16292246

[9] Secura GM, Allsworth JE, Madden T, Mullersman JL, Peipert JF. The Contraceptive CHOICE Project: reducing barriers to long-acting reversible contraception. Am J Obstet Gynecol. 2010;203:115.e1–7. 10.1016/j.ajog.2010.04.017 20541171 PMC2910826

[10] Trussell J. Contraceptive failure in the United States. Contraception. 2011;83:397–404. 10.1016/j.contraception.2011.01.021 21477680 PMC3638209

[11] Sedgh G, Singh S, Henshaw SK, Bankole A. Legal abortion worldwide in 2008: levels and recent trends. Int Perspect Sex Reprod Health. 2011;37:84–94. 10.1363/3708411 21757423

[12] Singh S, Sedgh G, Hussain R. Unintended pregnancy: worldwide levels, trends, and outcomes. Stud Fam Plann. 2010;41:241–50. 10.1111/j.1728-4465.2010.00250.x 21465725

[13] Cragan JD, Gilboa SM. Including prenatal diagnoses in birth defects monitoring: Experience of the Metropolitan Atlanta Congenital Defects Program. Birth Defects Res A Clin Mol Teratol. 2009;85:20–9. 10.1002/bdra.20508 19089857

[14] Centers for Disease Control and Prevention. Outcomes of pregnancies with laboratory evidence of possible Zika virus infection in the United States, 2016 [cited 2016 Jul 26]. https://www.cdc.gov/zika/geo/pregnancy-outcomes.html

[15] Grosse SD, Ouyang L, Collins JS, Green D, Dean JH, Stevenson RE. Economic evaluation of a neural tube defect recurrence-prevention program. Am J Prev Med. 2008;35:572–7. 10.1016/j.amepre.2008.07.008 18845415

[16] Trussell J, Hassan F, Lowin J, Law A, Filonenko A. Achieving cost-neutrality with long-acting reversible contraceptive methods. Contraception. 2015;91:49–56. 10.1016/j.contraception.2014.08.011 25282161 PMC4268022

[17] Truven Health Analytics. The cost of having a baby in the United States [cited 2016 May 16]. http://transform.childbirthconnection.org/wp-content/uploads/2013/01/Cost-of-Having-a-Baby1.pdf

[18] Biggio JR Jr, Morris TC, Owen J, Stringer JS. An outcomes analysis of five prenatal screening strategies for trisomy 21 in women younger than 35 years. Am J Obstet Gynecol. 2004;190:721–9. 10.1016/j.ajog.2003.09.028 15042005

[19] Santelli JS, Lindberg LD, Finer LB, Singh S. Explaining recent declines in adolescent pregnancy in the United States: the contribution of abstinence and improved contraceptive use. Am J Public Health. 2007;97:150–6. 10.2105/AJPH.2006.089169 17138906 PMC1716232

[20] Oduyebo T, Petersen EE, Rasmussen SA, Mead PS, Meaney-Delman D, Renquist CM, et al. Update: interim guidelines for health care providers caring for pregnant women and women of reproductive age with possible Zika virus exposure—United States, 2016. MMWR Morb Mortal Wkly Rep. 2016;65:122–7. 10.15585/mmwr.mm6505e2 26866840

[21] Fleming-Dutra KE, Nelson JM, Fischer M, Staples JE, Karwowski MP, Mead P, et al. Update: interim guidelines for health care providers caring for infants and children with possible Zika virus infection—United States, February 2016. MMWR Morb Mortal Wkly Rep. 2016;65:182–7. 10.15585/mmwr.mm6507e1 26914500

[22] Martines RB, Bhatnagar J, Keating MK, Silva-Flannery L, Muehlenbachs A, Gary J, et al. Notes from the field: evidence of Zika virus infection in brain and placental tissues from two congenitally infected newborns and two fetal losses—Brazil, 2015. MMWR Morb Mortal Wkly Rep. 2016;65:159–60. 10.15585/mmwr.mm6506e1 26890059

[23] Hamilton BE, Martin JA, Osterman MJ, Curtin SC, Matthews TJ. Births: Final Data for 2014. Natl Vital Stat Rep. 2015;64:1–64. 26727629

[24] Martin JA, Hamilton BE, Sutton PD, Ventura SJ, Menacker F, Munson ML. Births: final data for 2002. Natl Vital Stat Rep. 2003;52:1–113. 14717305

[25] Riehman KS, Sly DF, Soler H, Eberstein IW, Quadagno D, Harrison DF. Dual-method use among an ethnically diverse group of women at risk of HIV infection. Fam Plann Perspect. 1998;30:212–7. 10.2307/2991606 9782043

[26] Besnard M, Eyrolle-Guignot D, Guillemette-Artur P, Lastère S, Bost-Bezeaud F, Marcelis L, et al. Congenital cerebral malformations and dysfunction in fetuses and newborns following the 2013 to 2014 Zika virus epidemic in French Polynesia. Euro Surveill. 2016;21:30181. 10.2807/1560-7917.ES.2016.21.13.30181 27063794

[27] Alfaro-Murillo JA, Parpia AS, Fitzpatrick MC, Tamagnan JA, Medlock J, Ndeffo-Mbah ML, et al. A cost-effectiveness tool for informing policies on Zika virus control. PLoS Negl Trop Dis. 2016;10:e0004743. 10.1371/journal.pntd.0004743 27205899 PMC4874682

[28] Broyles RS, Tyson JE, Swint JM. Have Medicaid reimbursements been a credible measure of the cost of pediatric care? Pediatrics. 1997;99:E8. 10.1542/peds.99.3.e8 9099773

[29] Trussell J, Lalla AM, Doan QV, Reyes E, Pinto L, Gricar J. Cost effectiveness of contraceptives in the United States. Contraception. 2009;79:5–14. 10.1016/j.contraception.2008.08.003 19041435 PMC3638200

[30] Arroyo MP. Contraceptive access due to Zika threat. Elnuevodia [cited 2016 Aug 4]. http://www.elnuevodia.com/english/english/nota/contraceptiveaccessduetozikathreat-2227239

[31] Fowler C, Gable J, Wang J, Lasater B. Title X family planning annual report: 2014 national summary. Research Triangle Park (NC): RTI International; 2015.

[32] Portela M, Sommers BD. On the Outskirts of national health reform: a comparative assessment of health insurance and access to care in Puerto Rico and the United States. Milbank Q. 2015;93:584–608. 10.1111/1468-0009.12138 26350931 PMC4567854

[33] Bureau of Labor Statistics. May 2015 state occupational employment and wage estimates: Puerto Rico [cited 2016 Jul 20]. http://www.bls.gov/oes/current/oes_pr.htm

[34] Bureau of Economic Analysis. Table 2.5.4. Price indexes for personal consumption expenditures by function [cited 2016 Aug 5]. http://www.bea.gov/iTable/iTable.cfm?reqid=9&step=3&isuri=1&903=69#reqid=9&step=3&isuri=1&903=73

[35] Grosse SD. Assessing cost-effectiveness in healthcare: history of the $50,000 per QALY threshold. Expert Rev Pharmacoecon Outcomes Res. 2008;8:165–78. 10.1586/14737167.8.2.165 20528406

[36] McGann PT, Grosse SD, Santos B, de Oliveira V, Bernardino L, Kassebaum NJ, et al. A cost-effectiveness analysis of a pilot neonatal screening program for sickle cell anemia in the Republic of Angola. J Pediatr. 2015;167:1314–9. 10.1016/j.jpeds.2015.08.068 26477868 PMC4662897

[37] Burlone S, Edelman AB, Caughey AB, Trussell J, Dantas S, Rodriguez MI. Extending contraceptive coverage under the Affordable Care Act saves public funds. Contraception. 2013;87:143–8. 10.1016/j.contraception.2012.06.009 22840280 PMC5515367

[38] Frost JJ, Sonfield A, Zolna MR, Finer LB. Return on investment: a fuller assessment of the benefits and cost savings of the US publicly funded family planning program. Milbank Q. 2014;92:696–749. 10.1111/1468-0009.12080 25314928 PMC4266172

[39] Center for Work Force Studies. 2015 state physician workforce data book. Washington: Association of American Medical Colleges; 2015.

[40] Bishaw A, Fontenot K. Poverty: 2012 and 2013 [cited 2016 May 20]. https://www.census.gov/content/dam/Census/library/publications/2014/acs/acsbr13-01.pdf

