# Supplementary material for: Cost-effectiveness of Increasing Access to Contraception during the Zika Virus Outbreak, Puerto Rico, 2016
Source: Emerg Infect Dis. 2017 Jan;23(1):74–82. doi: 10.3201/eid2301.161322 (PMC5176229; doi:10.3201/eid2301.161322)
Supplement: Technical Appendix — Process for deriving the size of the target population, decision tree structure, and probabilistic sensitivity analysis of cost-effectiveness for a proposed intervention to increase access to contraception to women during the Zika virus outbreak, Puerto Rico, 2016. [file 16-1322-Techapp-s1.pdf]

Article DOI: http://dx.doi.org/10.3201/eid2301.161322

Cost-effectiveness of Increasing Access to Contraception during the Zika Virus Outbreak, Puerto Rico, 2016

Technical Appendix

Technical Appendix Table 1. Process for deriving the size of the target population for an intervention to increase access to contraception to women during the Zika virus outbreak, Puerto Rico, 2016*

Parameter	Estimate	Source
% Women of reproductive age in Puerto Rico at-risk for pregnancy† (A)	48.9%‡	(*1*)
% Women at risk for pregnancy using contraception (B)	88.1%‡	(*1*)
% Women not using permanent method (female or male sterilization) among contraception users (C)	48.8%	(*1*)
% Women of reproductive age in Puerto Rico at risk for pregnancy who are using nonpermanent contraception method (D)	21.0%	A × B × C
% Women at risk for pregnancy not using contraception (E)	11.9%	1 – B
% No contraception users who do not intend to become pregnant (F)	41.0%	Calculated based on 55% of births being unintended in Puerto Rico and the adjusted contraception use distribution in Puerto Rico reported in 2015
% Women who are at-risk for pregnancy, do not use contraception, and do not intend to become pregnant (G)	2.4%	A × F × E
% Target population among all women of reproductive age in Puerto Rico (H)	23.4%	D + G
Total population size of women of reproductive age in Puerto Rico in 2015 (I)	695,365	US Census Bureau§
Target population size	163,000 (rounded)	H × I

*Women of reproductive age in Puerto Rico who are sexually active with a male partner, fertile, not desiring pregnancy, and not using permanent contraception methods (e.g., tubal ligation and vasectomy)
†As in Bensyl et al. 2005 (*1*): women of reproductive age excluding persons who reported that they or their partner were pregnant, had a hysterectomy, were not sexually active, were too old to become pregnant, or were the same sex.
‡2015 contraception use distribution in Puerto Rico is based on 2002 contraception use distribution by adjusting the percentage of women of reproductive age in Puerto Rico at risk for pregnancy (subtract 7%) and the percentage of women at risk for pregnancy using contraception (add 4%). The assumptions are based on a 36% reduction of birth rate among women 15–44 years of age in Puerto Rico during 2002–2015, the decline in teen pregnancies was the fastest of any age group in Puerto Rico during 2010–2014, the reported reasons for US teen pregnancy reduction (a mixed of reduction in sexual activity and increased use of contraception), and Puerto Rico Title X clinics showing an increase of women using Title X services during 2005 and 2015.
§Data available at https://www.census.gov/popest/data.

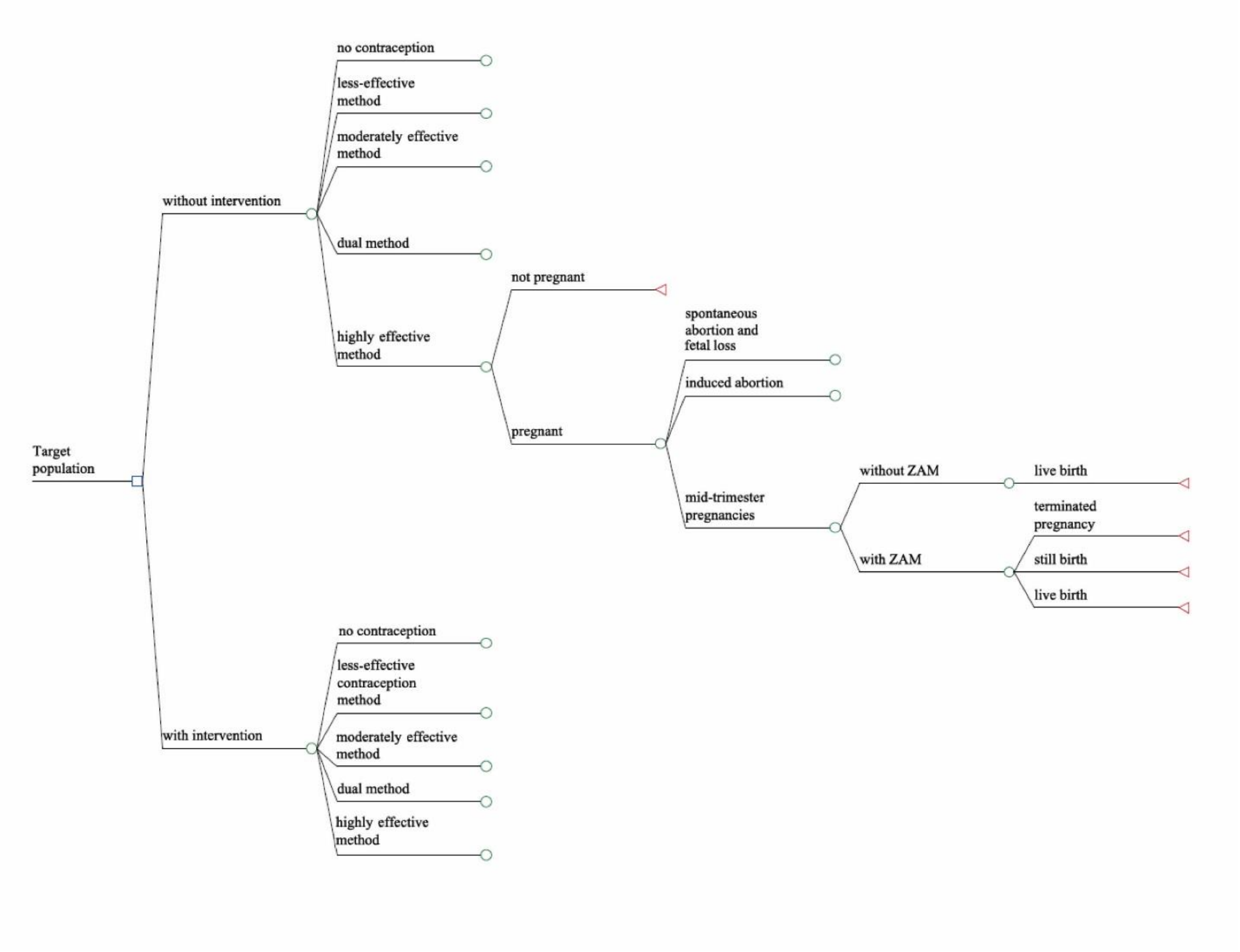

Technical Appendix Figure 1. Decision tree structure indicating contraceptive method use distribution, unintended pregnancy events, and the frequency of Zika virus–associated microcephaly for a cohort of 163,000 women at the time of an intervention to increase access to contraception to women during the Zika virus outbreak, Puerto Rico, 2016. ZAM, Zika virus–associated microcephaly.

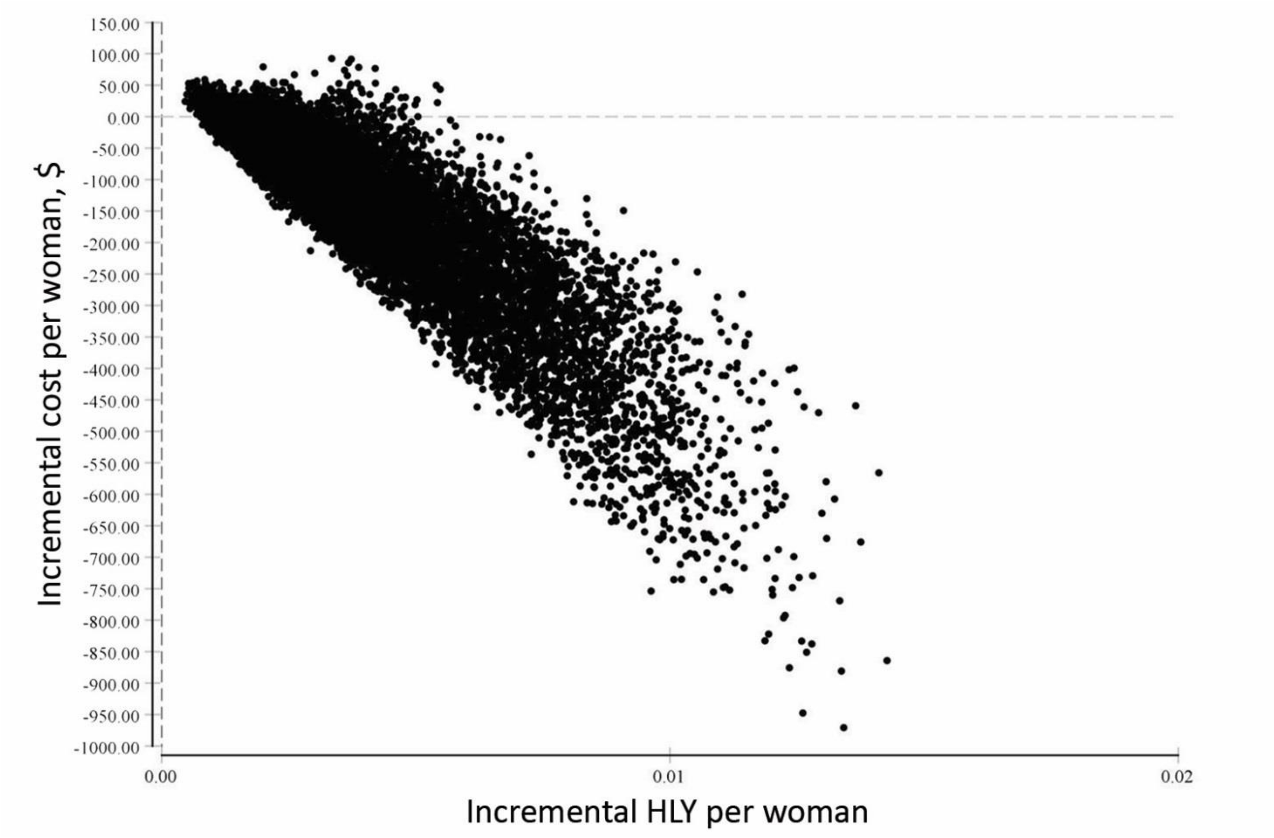

The vertical axis shows the incremental (difference of the) costs per woman between the scenarios with and without the intervention. The horizontal axis shows the incremental (difference of the) healthy life years per woman between the scenarios with and without the intervention. The dots represent the incremental cost-effective ratio of each iteration. The area below the dashed line indicates that in the iterations the “with intervention” scenario are cost-saving. HLY, healthy life years.

Reference

1. Bensyl DM, Iuliano DA, Carter M, Santelli J, Gilbert BC. Contraceptive use: United States and territories, behavioral risk factor surveillance system, 2002. MMWR Surveill Summ 2005;54:1–72. PubMed
